# Pneumomediastinum With Mediastinitis Following Third Molar Extraction With a High-Speed Air Handpiece: A Case Report and Literature Review

**DOI:** 10.7759/cureus.84695

**Published:** 2025-05-23

**Authors:** Hanna Sepsick, Boyu Ma, Qingcong Zeng, Jaime Castro-Núñez

**Affiliations:** 1 Dentistry, University of Alabama at Birmingham, Birmingham, USA; 2 Oral and Maxillofacial Surgery, University of Alabama at Birmingham, Birmingham, USA

**Keywords:** high-speed drill, impacted mandibular third molar, odontogenic mediastinitis, pneumomediastinum, third molar surgery

## Abstract

Pneumomediastinum and subcutaneous emphysema following third molar extraction are rare but potentially serious complications, often associated with the use of high-speed air-driven handpieces during dental procedures. This case report describes a 19-year-old male patient who developed pneumomediastinum, subcutaneous emphysema, deep neck, face, and mediastinal abscesses following wisdom tooth extraction with a high-speed air-driven handpiece, requiring urgent surgical intervention. The patient presented with worsening chest pain, voice changes, and facial swelling, with imaging confirming mediastinal air and abscess formation. Management included surgical incision and drainage (I&D) of the facial and neck abscesses and video-assisted thoracoscopic surgery (VATS) for the mediastinal abscess drainage, followed by admission to the intensive care unit. A review of literature from 2010 to 2025 emphasizes the rarity of this condition, with most cases linked to iatrogenic air introduction via high-speed handpieces. While the majority of cases resolve conservatively, severe complications such as mediastinitis or pneumothorax may require surgical intervention. Preventive strategies, including minimizing air-driven handpiece use and patient education on avoiding pressure-inducing activities, are crucial to reducing risk. This case highlights the importance of prompt recognition, advanced imaging, and tailored management to ensure favorable outcomes in this rare yet potentially life-threatening complication.

## Introduction

Pneumomediastinum, also known as mediastinal emphysema, is a rare but potentially life-threatening complication of third molar extraction, typically resulting from air infiltration through fascial planes due to high-speed air-driven handpieces [1]. Clinical symptoms range from subcutaneous crepitations, a crackling sensation on palpation, to localized swelling and more severe symptoms like chest pain, dyspnea, or voice changes when air reaches the mediastinum [2]. If the dissection extends into cervical or pharyngeal spaces and other danger spaces, patients may also experience neck pain, dysphagia, or odynophagia [3]. Symptom onset varies from hours to days post-procedure and is driven by the mechanism and extent of involvement. Severity spans from mild, self-limiting emphysema to critical complications, including pneumothorax, pneumopericardium, or mediastinitis, particularly when exacerbated by infectious processes [3,4].

High-speed air-driven handpieces, used for cooling and debris clearance during tooth sectioning, are the predominant culprits, inadvertently forcing air into soft tissues via extraction sockets or mucosal breaches [3,4]. Anatomically, mandibular third molars sit near the submandibular and sublingual spaces, offering a direct conduit for air to traverse deeper cervical, parapharyngeal, and retropharyngeal spaces into the mediastinum [5,6]. Maxillary third molar extractions, as in this case (teeth #1 and #16), are less prone to such deep air escape, making pneumomediastinum from this site less common [7]. Additional iatrogenic risks include excessive mucoperiosteal flap elevation or air-containing irrigants such as hydrogen peroxide. Post-operative behaviors that increase intrathoracic or oropharyngeal pressure, such as coughing, sneezing, or nose-blowing, may exacerbate air spread. Although exceedingly uncommon, non-iatrogenic etiologies, encompassing spontaneous or infectious origins, such as necrotizing fasciitis, are rare but possible [4,8,9].

The earliest documented case of subcutaneous emphysema following a dental procedure, reported in 1900 by Turnbull et al., described facial swelling subsequent to premolar extraction, likely exacerbated by the patient’s bugle-playing, which facilitated air dissection into the subcutaneous fascial planes [10]. Comprehensive literature reviews spanning 1960 to 2008, notably by Heyman and Babyof (1995) and McKenzie and Rosenberg (2000), cataloged 106 cases, predominantly associated with air-driven handpieces, compressed air syringes, and high-speed dental drills. These findings underscore the condition’s rarity and its intimate linkage with evolving dental technology [8,9]. 

Technological advancements in diagnostic imaging, particularly computed tomography (CT), have sharpened detection of these complications, as evidenced by Aslaner et al.’s reliance on CT to confirm air in subcutaneous and mediastinal spaces [3]. Nevertheless, the true incidence of these complications remains obscured by underreporting, attributable to asymptomatic presentations or diagnostic errors, frequently conflating the condition with angioedema or allergic reactions [3].

Management is guided by the severity of presentation. Mild cases confined to cervicofacial regions typically resolve within 7-10 days with conservative approaches: observation, analgesics, and instructions to avoid pressure-inducing activities [3,11]. Antibiotic prophylaxis, such as amoxicillin or amoxicillin-clavulanic acid, counters infection risks from oral flora dissemination through open extraction sites [3,11]. Mediastinal involvement warrants hospitalization and monitoring, occasionally supplemented by oxygen therapy to hasten air resorption via nitrogen gradient shifts [3]. Severe cases, marked by airway compromise, tension phenomena such as pneumothorax, or significant infection like mediastinitis, demand surgical intervention, ranging from abscess drainage to video-assisted thoracoscopic surgery (VATS), to stabilize patients and evacuate air or purulence [1,12].

This report describes a 19-year-old male patient who developed pneumomediastinum, subcutaneous emphysema, and mediastinitis with *Streptococcus constellatus* infection progressing to cardiogenic shock, 3-4 days post-maxillary third molar extraction (#1 and #16) performed using a high-speed air-driven handpiece. The clinical course necessitated multiple urgent surgical interventions. A comprehensive literature review spanning 2010 to 2025 follows, synthesizing risk factors, clinical presentations, and therapeutic approaches to inform preventive strategies and optimize management of this rare but potentially life-threatening complication.

## Case presentation

**Figure 1:**
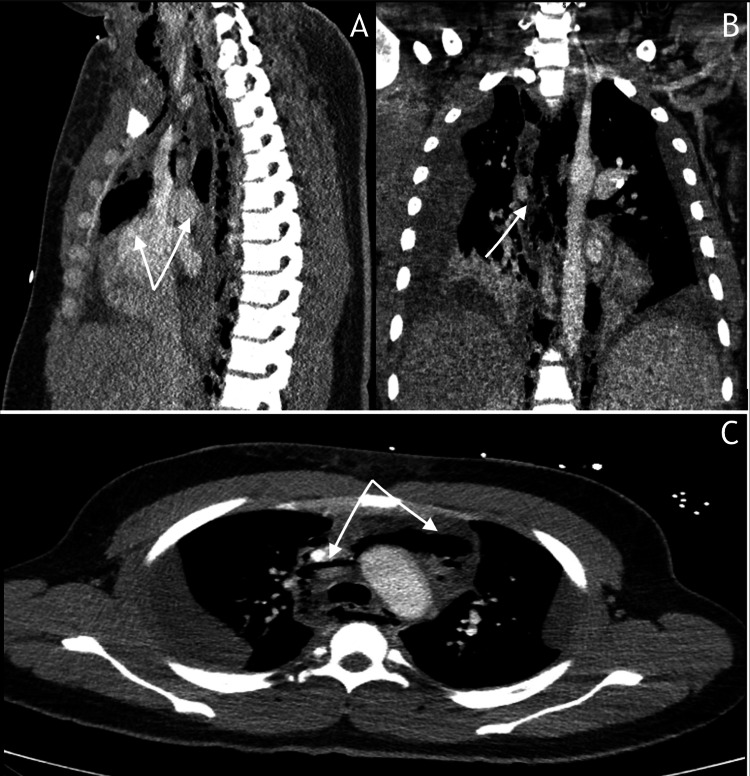
Initial computed tomography (CT) chest Imaging demonstrates pneumomediastinum and subcutaneous emphysema. (A) Sagittal view showing air tracking along fascial planes from the submandibular space to the superior mediastinum. (B) Coronal reconstruction reveals bilateral cervical subcutaneous emphysema extending to the thoracic inlet. (C) Axial section at the T3 level demonstrating mediastinal air surrounding the great vessels and trachea, with associated small bilateral pleural effusions

A 19-year-old male patient with no significant past medical history was transferred from an outside hospital (OSH) to the thoracic surgery service at the University of Alabama at Birmingham (UAB) Hospital for suspected esophageal perforation and mediastinitis. The patient had undergone extraction of the upper wisdom teeth one week prior, during which a high-speed air handpiece was utilized in a general dentist's office. Over the last 3-4 days, he had worsening chest pain, voice changes, and swelling around his neck. Post-procedure, he experienced worsening chest pain, voice changes, and facial swelling, which progressed to neck swelling over 3-4 days. At the OSH, a chest CT scan revealed mediastinal air, small bilateral pleural effusions, and subcutaneous air tracking into the neck and chest, prompting transfer to UAB for advanced care (Figure 1).

On arrival at the UAB’s emergency department, the patient exhibited mild respiratory distress and dyspnea, managed with 6 liters of oxygen via nasal cannula. Labs were significant for lactic acid (3.3 mmol/L), leukocytosis (15,000/mm^3^), respiratory rate of 30, and a maximum temperature of 102.4°F (Table 1). These trends satisfied the Systemic Inflammatory Response Syndrome criteria which is a checklist to follow a patient's vital signs to trend any signs of an inflammatory or infectious process. Elevated white blood cell count can also be a sign of infection. The patient was initially placed on meropenem and linezolid. The patient had tachypnea with a respiratory rate of around 30. Physical examination revealed crepitus in the neck and face, suggestive of subcutaneous emphysema, and a muffled voice. There was submandibular induration and swelling. He had trismus with an opening of 20 mm. The neck was also erythematous, extending down to the chest. Lactic acid trended to a peak of 4.4 mmol/L. Lactic acid trends can be an indication of increasing inflammation or infection. Given the concern for esophageal perforation, cellulitis, and descending pneumomediastinum, he was emergently taken to the operating room by the thoracic surgery and oral and maxillofacial surgery (OMFS) teams.

**Table 1:** Timeline of patient laboratory results Relevant labs including leukocytosis, lactic acid, temperature, respiratory rate, and microbial cultures related to the treatment of the patient

Blood tests	Initial exam admission day 1	Operative procedure 1-admission day 1	Admission day 3	Operative procedure 2-admission day 5	Operative procedure 3- admission day 8	Admission day 15	Admission day 30-labs	Reference range
Leukocytosis	15,220/mm^3^	22,100/mm^3^	24,680/mm^3^	45,590/mm^3^	31,060/mm^3^	14,650/mm^3^	8,000/mm^3^	4,500-11,000/mm^3 ^[13]
Lactic acid	3.3 mmol/L	4.4 mmol/L	1.7 mmol/L	2.4 mmol/L	N/A	N/A	N/A	0.5-2.2 mmol/L [14]
Temperature	102.4°F	102.7°F	102.2°F	102.4°F	99.8°F	99.8°F	99.6°F	97-99°F [15]
Respiratory rate	30 breaths/min	Ventilator	Ventilator	Ventilator	Ventilator	Ventilator	Trach collar	12-18 breaths/min [16]
Microbial cultures/antibiotics	Meropenem/linezolid	Meropenem/linezolid	*Streptococcus constellatus *(+), meropenem/linezolid	Meropenem/linezolid	Transitioned to meropenem/ceftriaxone, sensitive to cephalosporins		Finished 4 weeks of ceftriaxone	

In the operating room, the patient was planned for an incision and drainage (I&D) of a facial abscess by OMFS and right VATS with drainage of a mediastinal abscess by thoracic surgery. VATS was selected for its minimally invasive approach, eliminating the need for a fully open thoracotomy [17]. VATS has been demonstrated to result in shorter hospital stays, reduced incidence of post-operative pneumonia, and decreased requirements for prolonged intubation and transfusions [18]. He was intubated with no complications and remained on vasopressors through the procedure. Afterward, the patient was prepped and draped in a sterile fashion in a supine position. A 3 cm incision was made in the submandibular region. Blunt dissection was used to enter the right submandibular space, sublingual, submental space, buccal space, lateral pterygoid space, and the sublingual space. Purulent fluid was encountered, and cultures were sent. The wound was irrigated multiple times with copious normal saline, and four drains were placed into the aforementioned spaces.

**Figure 2:**
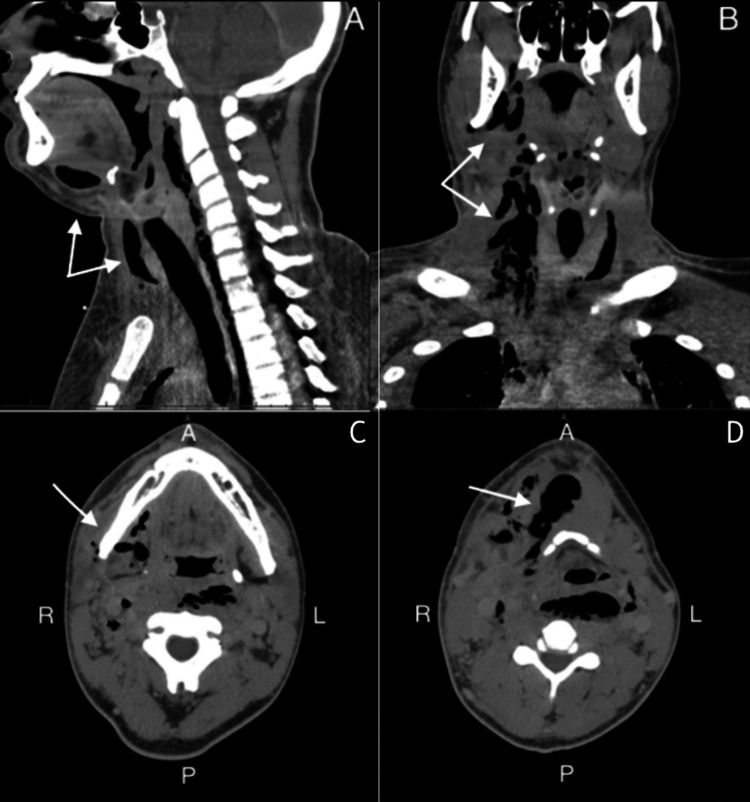
Cross-sectional CT characterization of air dissection patterns CT: computed tomography (A) Sagittal view at the hyoid level showing extensive subcutaneous emphysema in the superficial and deep cervical spaces. (B) Coronal reconstruction illustrates the craniocaudal extent of mediastinal emphysema, with air dissecting along the tracheoesophageal bundle into the anterior mediastinum. Note the preservation of esophageal wall integrity. (C) Axial view at the level of the mandible demonstrating a localized mediastinal abscess. (D) Axial view at the level of the hyoid bone demonstrating a localized mediastinal abscess

Thoracic surgery made incisions around the seventh intercostal space, posteriorly approximately one fingerbreadth below and posterior to the tip of the scapula, and anterior to the chest wall. Purulent drainage was seen from the mediastinum with thickened mediastinal pleura. The mediastinal pleura was accessed, and purulent fluid was evacuated from the region, followed by the placement of a 24-French Blake drain. Intraoperative findings confirmed subcutaneous emphysema with purulent drainage extending from the face to the chest, mediastinal air, and a localized mediastinal abscess without esophageal perforation (Figure 2). Drains included three chest tubes (one Blake drain) and four Penrose drains (two in the right neck, one in the submental space, and one in the submandibular space).

**Figure 3:**
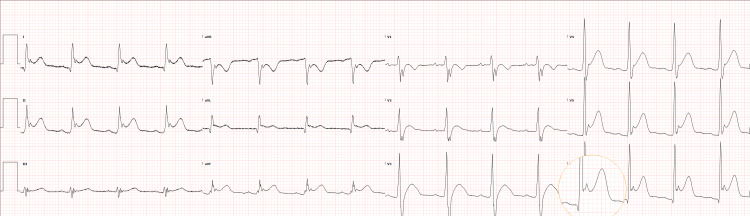
Electrocardiographic manifestations of mediastinal infection ECG: electrocardiogram Twelve-lead ECG obtained during hemodynamic instability shows diffuse ST-segment abnormalities (leads I, II, aVF, V3-V6) with reciprocal PR-segment depression, consistent with pericardial inflammation. These findings, in the context of progressive mediastinitis, prompted evaluation for septic cardiomyopathy and infectious pericarditis. The circle shows PR depression and ST elevation

**Figure 4:**
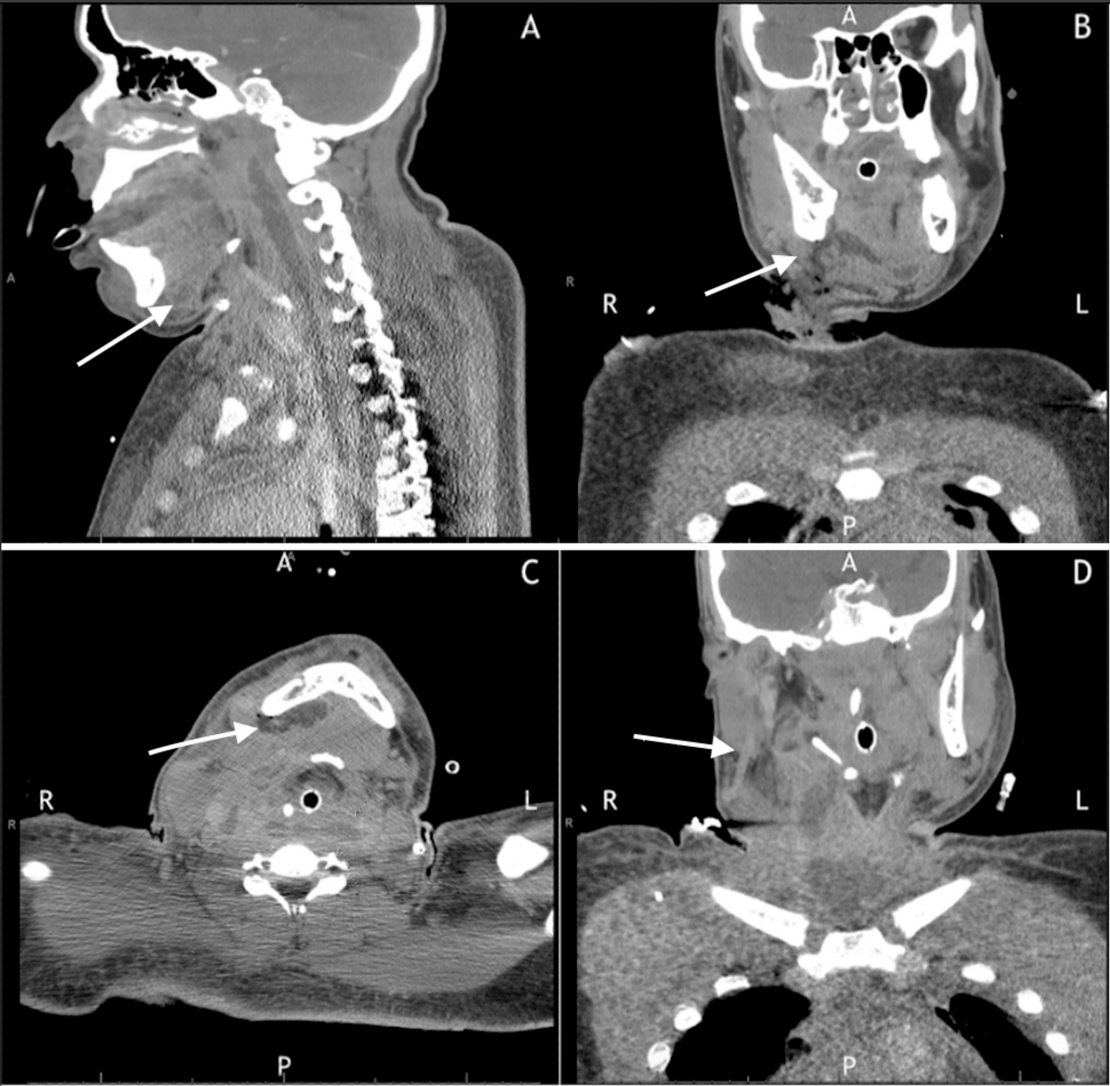
CT scan after surgical intervention CT: Computed tomography Evolution to mediastinal abscess on follow-up imaging. (A) Sagittal contrast-enhanced CT neck demonstrating organized fluid collections with enhancing walls in the mediastinum, representing abscess formation. (B) Coronal view showing residual cervical emphysema with new rim-enhancing collections along the surgical drainage pathways. Note the developing pleural reaction and residual pneumomediastinum. (C) Axial view further demonstrating fluid collection representing abscess formation. (D) Coronal view showing the posterior extent of the abscess near the mandible

Post-operatively, the patient was admitted to the surgical intensive care unit (SICU), intubated, and sedated. The patient was placed on vancomycin, cefepime, and micafungin empirically. Troponin elevated to 31,000 mm^3^ on 3/26 with the electrocardiogram (ECG) showing diffuse ST changes with PR depressions (Figure 3). The clinical findings suggested cardiogenic shock, necessitating urgent consultation with cardiology to orchestrate precise management of the cardiovascular instability. Concurrently, infectious disease expertise was sought to direct tailored antimicrobial therapy. Microbial cultures taken from the operating room grew *Streptococcus constellatus*. This gram-positive microorganism, a constituent of the normal oral flora, is frequently implicated in purulent head and neck infections upon breaching the confines of the oral cavity. The patient continued to have elevated leukocytosis in the 30,000/mm^3^ peaking up to 45,000/mm^3^. Subsequent imaging with contrast-enhanced CT of the neck and chest revealed multiple fluid collections in the cervical region and anterior mediastinum (Figure 4).

Due to the presence of a fluid collection with concerns for a lingering infection, the patient was taken back to the operating room by thoracic surgery and OMFS for a second I&D procedure on admission day 5. The transcervical incision was extended posteriorly toward the mastoid process to delineate the sternocleidomastoid muscle. Dissection advanced to the clavicle, encountering profuse purulent exudate at the mediastinal site previously explored via mediastinoscopy. Penrose drains were then placed deep within the sternocleidomastoid compartment.

The patient continued to exhibit leukocytosis, peaking at 29,000/mm³ and remaining elevated around 28,700/mm³, accompanied by high-grade fevers (Table 1). On admission day 8, these findings with concern for a systemic response due to a residual infection prompted a return to the operating room with both the OMFS and thoracic surgery teams. The anterior mediastinum was re-accessed and irrigated. Additionally, the parapharyngeal and retropharyngeal spaces were re-explored to disrupt loculations. Following this procedure, the cultures grew sensitive to cephalosporins, and the patient was transitioned to ceftriaxone and kept on meropenem. Meropenem was discontinued on post-operative day 19, and the patient was kept on ceftriaxone for four weeks total. 

The patient began to show clinical improvement, with a gradual decline in leukocytosis over the subsequent two weeks. The submandibular swelling decreased, and induration improved by the first post-operative week. Two weeks into his initial admission, the patient had a tracheostomy done due to the risk of tracheal stenosis with long-term ventilator dependence and endotracheal intubation. There were no long-term sequelae from the tracheostomy site, and the wound bed closed once the patient was decannulated. The patient was weaned off the ventilator four weeks post-operatively, and then the patient’s tracheostomy was downsized and decannulated once the patient was stable on room air. Initially, he demonstrated a limited oral opening of 25 mm, likely secondary to prolonged intubation. Limited mouth opening could have been worsened by myositis from the infection and atrophy of the muscles due to inability to open and close while intubated. By the time of discharge, he was decannulated, and his swelling had mostly resolved. He remained afebrile, with a normal heart rate and blood pressure, and a normal respiratory rate. At his two-week follow-up visit post-discharge, his maximal incisal opening had improved to 30 mm. He was tolerating a regular diet and exhibited no cranial nerve deficits.

## Discussion

Pneumomediastinum and subcutaneous emphysema following third molar extraction arise from air infiltrating soft tissue planes, typically via high-speed air-driven handpieces, as occurred in this patient’s extraction of maxillary third molars (#1 and #16) [4]. While mandibular molar roots commonly abut the submandibular space, facilitating air transit through parapharyngeal and retropharyngeal spaces to the mediastinum, accounting for approximately one-third of cases per Sood et al. [19]. In contrast, this case’s air originates from the maxillary spaces (e.g., buccal, pterygoid), representing an uncommon pathway. The high-speed handpiece likely initiated air entry, with subsequent abscess formation signaling an infectious component. This contrasts with Peters et al.’s turbine-free case implicating patient-induced pressure (e.g., Valsalva maneuver) [4]. Together, these findings highlight the potential for iatrogenic factors in the pathogenesis of post-extraction pneumomediastinum.

Literature reviews by Heyman and Babayof (1995) and McKenzie and Rosenberg (2009), spanning 1960 to 2008, documented 106 cases of subcutaneous emphysema, pneumomediastinum, and pneumothorax, primarily linked to air-driven handpieces, compressed air syringes, and high-speed dental drills [8,9]. Heyman and Babayof reported two cases requiring surgical intervention: one post-amalgam restoration necessitating tracheostomy and intensive care for subcutaneous, retropharyngeal, and mediastinal emphysema, and another following dental extraction requiring thoracic drainage and peritoneal decompression for pneumomediastinum and pneumothorax [20,21]. Infections complicated three cases, with two at dental sites and one fatal cervical subcutaneous emphysema case due to disseminated infection, possibly from gas-forming bacteria, as seen in a submandibular swelling case tied to a mandibular tooth infection [21-23]. McKenzie and Rosenberg described two surgical cases: a 45-year-old woman post-apicoectomy needing exploratory laparotomy, bilateral chest tubes, and mechanical ventilation for pneumomediastinum and pneumothorax exacerbated by vomiting, and another requiring emergency tracheotomy and chest tube placement for airway obstruction and pneumothorax [24,25].

To evaluate the incidence of post-extraction pneumomediastinum from this time period on, a comprehensive literature review was conducted utilizing Scopus, Google Scholar, and PubMed from 2010 to 2025 to identify scientific articles detailing cases of pneumomediastinum arising from a handpiece. The search employed keywords such as “pneumomediastinum," "pneumomediastinum and handpiece,” "pneumomediastinum and extraction," "pneumomediastinum and dental extraction," "subcutaneous emphysema and extraction," and "subcutaneous emphysema and handpiece." Inclusion criteria were restricted to studies published in English that described pneumomediastinum arising from a handpiece. The search strategy yielded six studies, comprising six case series/retrospective studies.

Pneumomediastinum after dental extraction is exceptionally rare. Peters et al. identified 26 cases between 2010 and 2020, while Tegenbosch et al. report 44 from 1973 to 2023 [4,26]. This is sparse relative to the millions of annual extractions performed. Guillén-Paredes et al. found no instances in 100 extractions (<1% incidence), a scarcity Ye et al. also echo with mediastinal involvement as even rarer [27,28]. Underreporting likely skews prevalence estimates, as asymptomatic cases, like Guillén-Paredes et al.’s painless case example, or misdiagnosed symptomatic cases evade detection [27]. The current case, marked by extensive mediastinal spread and requiring multiple surgical interventions, represents a severe outlier on the clinical spectrum, which ranges from subclinical to life-threatening presentations.

Although mortality from pneumomediastinum post-extraction remains negligible across studies, morbidity varies widely. Most cases resolve conservatively within 2-10 days, with Tegenbosch et al. citing 2-7 days for isolated subcutaneous emphysema and Peters et al. reporting 80% resolution by day 9-10 [4,26]. Severe complications- pneumothorax (11% in Peters et al.), pneumopericardium (7%), or mediastinitis- elevate morbidity, as seen in this patient [4,26]. Dirol et al. estimate a 40% mediastinitis risk in iatrogenic cases, yet dental instances rarely escalate to this degree. Here, abscess development, cardiac shock (troponin 31,000 mm^3^), and a prolonged two-week recovery highlight exceptional morbidity, mitigated by robust intervention [29].

High-speed air-driven handpieces, implicated in 65% of Peters et al.'s cases and in this patient’s procedure, pose a primary risk by forcing air into tissue planes, particularly near mandibular molars, which is reported in 63.6% of cases per Tegenbosch et al. [4,26]. Ye et al. and Tegenbosch et al. corroborate this, though this maxillary case broadens the anatomical scope [26,28]. Surgical factors such as excessive flap elevation or bone defects, alongside impacted teeth, amplify vulnerability [28]. Patient actions such as coughing may contribute, but this case’s delayed onset (3-4 days) and absence of such triggers point to an iatrogenic origin tied to the dental handpiece [28].

Bacterial involvement significantly exacerbates the severity of pneumomediastinum, as exemplified by the isolation of *Streptococcus constellatus*, a member of the *Streptococcus anginosus* group known for its propensity to form abscesses and precipitate invasive infections, from the initial surgical intervention in this patient. This pathogen precipitated mediastinitis, necessitating multiple surgical interventions and aggressive antimicrobial therapy, starkly contrasting with the typically sterile nature of other pneumomediastinum cases. Aslaner et al. highlight the risk of oral flora contamination, including pathogens such as *Legionella *and *Pseudomonas*, advocating for broad-spectrum antibiotics like ampicillin-sulbactam to mitigate infection risks from contaminated air introduced during dental procedures [3]. Similarly, Peters et al. document the prophylactic use of amoxicillin-clavulanic acid in 23 of 26 cases, despite the absence of specific pathogen identification, underscoring a preventive approach to microbial complications [4].

Yang et al. emphasize the potential for contamination during dental procedures, yet the patient’s *Streptococcus constellatus* infection, marked by profound leukocytosis (45,000/mm³) and cardiac shock (troponin 31,000 mm³), represents an exceptionally severe clinical manifestation [30]. This virulence likely stemmed from breaches in sterile technique or delayed presentation (3-4 days), which facilitated bacterial seeding and dissemination. The resultant infection necessitated an intensive antimicrobial regimen, including vancomycin, cefepime, and micafungin, alongside surgical debridement to address the mediastinal and subcutaneous gas collections. Kannangara et al.’s analysis of 61 pyogenic dental infections identified *Staphylococcus epidermidis* as the predominant aerobic isolate, followed by *Streptococcus *species, highlighting the microbial diversity in such infections [31]. Bartlett et al.’s study of 15 space infections along the mandible and neck further delineates the role of anaerobes, noting clinical hallmarks such as gas in tissues, foul-smelling exudate, and Gram staining of exudates as presumptive indicators of anaerobic infection [32]. Among these, anaerobic streptococci, including *Streptococcus constellatus* as observed in this case, are principal pathogens in head and neck anaerobic infections, underscoring their potential to drive severe, life-threatening complications when sterile barriers are compromised.

Peters et al. report 26 cases, 77% with pneumomediastinum, 65% turbine-related, with common symptoms like crepitus (96%) matching this case. On the other hand, the patient developed mediastinitis and cardiac sequelae, which are atypical [4]. Ye et al.’s turbine cases resolved in 3-7 days with antibiotics, Guillén-Paredes et al.’s asymptomatic case in four days, and Tegenbosch et al.’s dysphagia case in seven, all contrasting this patient’s multisurgical, prolonged course [26-28]. Diagnosis relies on clinical findings (crepitus, swelling) and imaging, which in this case delineated emphysema extent and complications (e.g., abscesses) from other potential diagnoses (e.g., hematoma, allergic reactions, and cellulitis) [4,26]. Management spans from conservative (antibiotics, observation) to surgical (I&D, VATS) and SICU care based on severity, reflecting mediastinal criticality and aligning with literature advocating hospitalization [4,26,28].

Preventive measures to curb iatrogenic air entry include minimizing high-speed air-driven handpiece use, favoring surgical handpieces with sterile irrigation, and limiting mucoperiosteal flap detachment [4,19,26,28,33]. Post-operative instructions should advise against activities increasing intraoral pressure (e.g., blowing nose, smoking), though this case lacked such triggers, suggesting iatrogenic air introduction as the primary cause. Ye et al. emphasize pre-surgical counseling for impacted teeth, which supports risk mitigation in such rare, severe presentations [28], and pre-operative counseling for patients who are at higher risk of infection, such as those who are immunocompromised. A course of antibiotics could also be suggested. Furthermore, as the cause of these cases is due more to poor operative choice of handpiece, it may be important to educate general dentists on the use of high-speed air-driven handpieces during extraction to avoid such a severe presentation. Education could be in the form of lectures or regulations regarding the use of specific handpieces for certain procedures. Furthermore, analyzing trends such as lactic acid, white blood cell count, and vital signs, including temperature, helps elucidate any signs of an inflammatory process.

## Conclusions

This case illuminates the rare but grave risk of pneumomediastinum with mediastinitis post-third molar extraction using a high-speed air handpiece, escalating from air infiltration to *Streptococcus constellatus*-driven abscesses, cardiac shock, and a two-week recovery requiring three surgical interventions (VATS, I&D). While the literature shows most cases resolve conservatively within days, this patient’s severe morbidity underscores the need for early recognition via CT, critical for detecting fluid collections, and prompt, aggressive management, including tailored antibiotics and surgery. Preventive strategies, including judicious air-driven handpiece use, meticulous technique, and patient education on avoiding pressure-inducing acts, are paramount, particularly for maxillary extractions with delayed infectious potential. Clinicians must maintain heightened suspicion for diagnostic symptoms, such as chest pain, voice changes, and fever, to avert such life-threatening outcomes.
